# Oncologic Imaging and Radiomics: A Walkthrough Review of Methodological Challenges

**DOI:** 10.3390/cancers14194871

**Published:** 2022-10-05

**Authors:** Arnaldo Stanzione, Renato Cuocolo, Lorenzo Ugga, Francesco Verde, Valeria Romeo, Arturo Brunetti, Simone Maurea

**Affiliations:** 1 Department of Advanced Biomedical Sciences, University of Naples “Federico II”, 80131 Naples, Italy; 2 Department of Medicine, Surgery and Dentistry, University of Salerno, 84081 Baronissi, Italy

**Keywords:** radiomics, reproducibility, oncologic imaging, evidence-based medicine, research quality

## Abstract

**Simple Summary:**

Radiomics could increase the value of medical images for oncologic patients, allowing for the identification of novel imaging biomarkers and building prediction models. Unfortunately, despite the many promises and encouraging findings, the translation of radiomics into clinical practice appears as a distant goal. Indeed, challenges such as generalizability and reproducibility are slowing down the process and must be faced with a rigorous and robust radiomic methodology. In this review, we turn the spotlight to the methodological complexity of radiomics, providing an outline of dos and don’ts aimed at facilitating state-of-the-art research.

**Abstract:**

Imaging plays a crucial role in the management of oncologic patients, from the initial diagnosis to staging and treatment response monitoring. Recently, it has been suggested that its importance could be further increased by accessing a new layer of previously hidden quantitative data at the pixel level. Using a multi-step process, radiomics extracts potential biomarkers from medical images that could power decision support tools. Despite the growing interest and rising number of research articles being published, radiomics is still far from fulfilling its promise of guiding oncologic imaging toward personalized medicine. This is, at least partly, due to the heterogeneous methodological quality in radiomic research, caused by the complexity of the analysis pipelines. In this review, we aim to disentangle this complexity with a stepwise approach. Specifically, we focus on challenges to face during image preprocessing and segmentation, how to handle imbalanced classes and avoid information leaks, as well as strategies for the proper validation of findings.

## 1. Introduction

**Figure 1:**
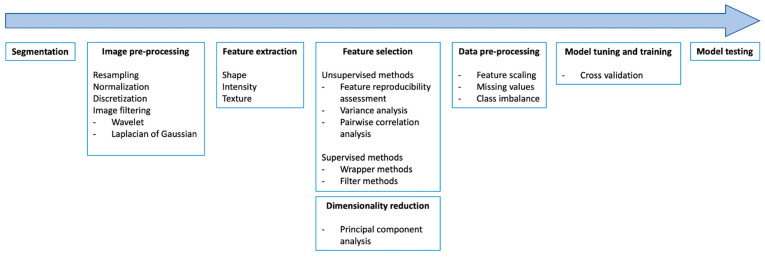
The main steps of a radiomic pipeline.

Historically, medical images have been evaluated visually and radiologists interpreted imaging findings and generated reports using semantic descriptors. Unfortunately, there are intrinsic limitations to this approach, which is prone to subjectivity and thus interobserver variability. Semiquantitative and quantitative approaches to diagnostic imaging have gradually emerged to further increase its value, especially in oncology [1]. More recently, advancements in medical imaging equipment, the digitalization of diagnostic images, and the high computational power available today have made it possible to obtain additional information from imaging scans, far beyond what meets the eyes of radiologists [2]. Indeed, medical images can be turned into mineable data, extracting numerous quantitative parameters to generate high-dimensional data with a complex, multi-step process known as radiomics. This postprocessing technique allows for the characterization of image heterogeneity at the pixel level, which is supposed to reflect biological heterogeneity, a peculiarity of cancer. Following this assumption, radiomics appears as a promising evolution in the field of oncologic imaging, and the scientific community has been tirelessly working to provide the evidence necessary for its possible translation into clinical practice, with a constant rise in the number of publications over time [3]. Radiomics could unveil novel imaging biomarkers or be paired with machine learning (ML) to train support decision tools and classifiers to aid physicians in the management of oncologic patients. However, the actual applications of radiomics to the benefit of oncologic patients are still a distant goal, and some suggest that methodological quality has not been rising along with quantity, with many studies presenting methodological shortcomings in their radiomic pipelines [4,5,6]. Due to the complexity of radiomic pipelines (Figure 1), the scientific community is facing reproducibility and generalizability issues [7]. With the present review, we aim to provide an overview of the technical challenges that need to be tackled in order to produce robust evidence and foster the translation of radiomics into clinical practice. In particular, we focus on hand-crafted radiomics and ML approaches, with minor references to the world of deep learning (DL).

## 2. Image Preprocessing and Quality Control of Imaging Datasets

One of the most interesting aspects and a great advantage of radiomics is that it can be applied to routinely acquired medical images. Thus, the development and deployment of radiomic approaches do not negatively impact the current radiological workflow [8]. However, while many imaging protocols and technical acquisition parameters have been standardized in the field of oncologic imaging, there are significant sources of variability that may affect the reproducibility of the computational biomarkers known as radiomic features to a relevant extent. Indeed, non-biological variations can be introduced by differences in terms of scanner characteristics (e.g., manufacturer, model, hardware components) as well as imaging protocols, with the adherence to imaging guidelines recommendations on technical acquisition parameters being rather heterogeneous [9,10,11]. Furthermore, the variability of image reconstruction algorithms and/or their settings might generate noise in the form of spurious variations in certain radiomic features, making it necessary to identify such unstable features so that they be excluded from further analysis [12,13,14]. The test–retest analyses of radiomic feature robustness have shown that the number of stable features might be low and possibly vary in different organs [15,16]. In this light, it appears evident that well-recognized issues with imaging biomarkers such as repeatability (multiple measurements with the same equipment in the same subject) and reproducibility (measurements performed with different equipment) apply to radiomics as well [17]. To account for these issues, there is a variety of countermeasures that collectively constitute image preprocessing techniques. These aim to homogenize medical images with respect to those variables (e.g., pixel spacing, grey-level intensities, bin width) that could generate a non-biologically relevant and noisy heterogeneity [15]. A summary of the main image preprocessing techniques can be found in Table 1. It deserves to be underlined that the test–retest robustness of radiomic features is significantly affected by the image preprocessing implementation or lack thereof [18,19]. Even more interestingly, it has been found that the impact of different image preprocessing procedures may differ depending on the nature of medical images. This was reported, for example, in a test–retest breast cancer MRI study carried out by Grazier et al. in which stable features varied based on MRI sequence [20]. In a different oncologic setting, namely prostate cancer, it was similarly found that both image type and preprocessing can greatly affect the repeatability of radiomic features [21]. As a consequence, it is of paramount importance to disclose all the information regarding the image preprocessing in detail, so that the findings of radiomic studies can be properly replicated and eventually validated.

It should be also acknowledged that high-quality, standardized medical images can help ensure accurate, reproducible, and repeatable results [22]. In this setting, publicly available imaging datasets represent a valuable asset, with the dual benefit of increasing data openness while allowing researchers to access verified medical images from high volumes and highly specialized centers. However, even widely embraced public datasets should be subject to independent quality controls since the occurrence of shortcomings has been highlighted [23,24]. Among the issues that can reduce the quality of medical images, motion artifacts represent a common problem that could be faced with image preprocessing with motion correction, although this might introduce a potential variation in radiomic data; thus, motion-control strategies (e.g., breath holding) should be probably preferred, while one might want to entirely exclude images with artifacts from the dataset [25].

## 3. Lesion Segmentation

**Figure 2:**
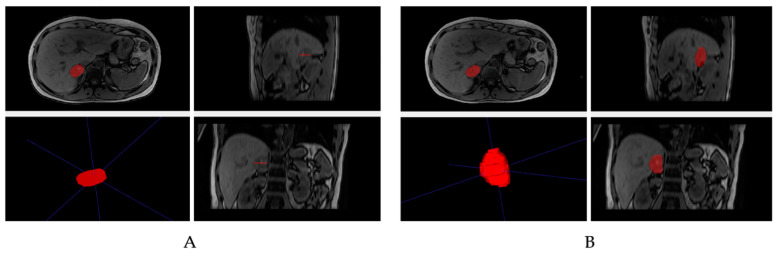
In this example, two-dimensional (**A**) and three-dimensional (**B**) approaches for adrenal lesion segmentation on magnetic resonance images are shown.

In the computer vision field, segmentation is the process of clustering the parts of an image that belong to the same object class together. In the development of a radiomic workflow applied to oncological imaging, segmentation represents a crucial step to define the tumoral region of interest from which the imaging data are extracted and computed into the machine-readable and quantitative features necessary for the subsequent radiomic analysis. The boundaries of a tumoral lesion may be delineated in two-dimensional or three-dimensional approaches, thus generating a region of interest (ROI) or a volume of interest (VOI), respectively (Figure 2).

Segmentation methods can be classified as manual, semiautomatic, and automatic, and each method offers advantages and disadvantages [26]. Manual segmentation is performed in a hand-drawing manner using a mouse or a graphic tablet with the advantage of precise definitions for ROIs and/or VOIs, especially when applied to small-size datasets by expert radiologists. On the other hand, this method involves time-consuming procedures and may suffer from high intra- and interobserver variability, thus introducing bias in the results of radiomic pipelines [27]. Semiautomatic segmentation consists of the user-guided application of algorithms that adopt different strategies of image delineation such as region-growing, level set, graph cut, and active contour (snake) approaches [12]. Although this method reduces labor tasks and may increase radiomic feature robustness in comparison to an entirely manual segmentation process [28], the stability of radiomic models still remains susceptible to subjective bias, especially in the case of intensive user correction. Recently, fully automatic systems based on DL architecture such as convolutional neural networks (CNNs) have been applied to medical imaging segmentation showing excellent results in lesion detection and segmentation and avoiding the intra- and interobserver variability of radiomic features due to the deterministic nature of the CNNs’ output [29]. The main drawbacks of DL-based automatic methods are related to their need for large-sized labeled data to train an accurate model and the generalizability of trained algorithms since they might perform very poorly on a dataset different from that of training [30]. Interestingly, while these DL approaches could be of aid to reduce the workload and increase reproducibility in the setting of hand-crafted radiomic research, when used for classification purposes, DL models do not require image segmentation.

Tumor segmentation entails the main challenge of radiomic feature reliability, especially for manual and semiautomatic methods, given the lack of standardized segmentation procedures, which may lead to the poorly reproducible application of these models [31]. Indeed, emerging exploratory works aimed to assess to what degree radiomic feature robustness can be influenced by segmentation variability in terms of margin variations [32,33], tumor site [34], the choice of region or volume of interest [35], and the use of manual or (semi)automatic methods [36].

In this context, the influence of segmentation interobserver variability on radiomic features stability has been evaluated by Haarburger et al. [37] on three large publicly available datasets of CT images across three tumor sites, namely the lung, kidney, and liver. Manual segmentation by four expert radiologists was performed on the dataset of lung tumors, whereas twenty-five different segmentations were computed using an automatic method relying on a DL neural network on all three datasets. They found comparable results between the manual and automatic methods, while their reproducibility analysis showed a significant difference between feature categories, with shape features resulting in the most stable features across the different methods and datasets. The reproducibility assessment of features across segmentations can be quantified through the intraclass correlation coefficient (ICC) ranging from 0 to 1, with a value below 0.5 indicating a poor agreement between raters on a radiomic feature [38]. In two recent systematic reviews assessing the methodological quality of radiomic studies on prostate MRI [39] and adrenal CT/MR imaging [5], it was revealed that most of the analyzed studies (85% for prostate MRI radiomics and 72% for adrenal radiomics) suffered from a lack of feature stability to multiple segmentation testing, underlining the need for more robust radiomic pipelines.

Efforts in providing a comprehensive overview of features stability techniques have been made by Kocak et al. [40] with a systematic review focused on the reproducibility strategies adopted in the radiomic studies of renal lesions, showing that segmentation variability analysis was largely implemented in comparison to image acquisition or the analysis of processing differences. Nevertheless, a reproducibility analysis based on segmentation variability was evaluated only in less than half of the included works (18/41). Similarly, Gitto et al. [41] systematically reviewed feature reproducibility issues in the studies focused on CT and MRI radiomics of bone and soft-tissue sarcomas, pointing out that interobserver segmentation variability was applied only in 33% of investigations. Consensus guidelines and standardized procedures are needed in segmentation step analysis for the profiling of reproducible radiomic pipeline to finally move oncological imaging toward an integrated biological and computational data science providing high-quality research for real clinical uses.

## 4. Feature Extraction and Selection

The term biomarker refers to a measurable indicator of a specific biological state. Imaging biomarkers can be both qualitative, i.e., based on an interpretation of experts, and quantitative, extrapolated through mathematical formulas. In particular, radiomic features characterize the regions or volumes of interest that have been previously identified [42]. Unfortunately, the lack of reproducibility and validation of these high-throughput image biomarkers undermines the possible clinical implementation of this approach. In recent years, with the significant increase in radiomic studies and applications, a shared document concerning the standardization of feature extraction has become necessary. In this scenario, the image biomarker standardization initiative (IBSI) was born, an international collaborative project focused on the definition of guidelines concerning radiomic quantitative feature extraction [43]. The drafted document, which can be consulted on the official website of the initiative (https://ibsi.readthedocs.io, accessed on 29 August 2022), describes intensity-based statistical features, intensity histogram features, intensity-volume histogram features, morphological features, local intensity, and textural matrix-based features, as well as how to report textural parameters. Considering the availability of these guidelines, all radiomic studies should conform to them, in order to ensure the possible reproducibility of the obtained results. In addition to extraction, another crucial step in the radiomic workflow is the identification of a subset of relevant features through feature selection. Indeed, using an ML approach, it is desirable to reduce the number of input variables to both reduce the modeling computational burden and improve the algorithm performance when developing a predictive model. In this setting, feature selection is also crucial to prevent overfitting, i.e., the production of a model too closely matching the training and validation data, which fails to fit new data after clinical implementation. In the face of a plethora of features, this step can be achieved by using statistical-based feature selection methods. These approaches include unsupervised methods, blind to the target variable, and supervised techniques, which select those features presenting the strongest relationship with the output variable [44].

The main unsupervised methods comprise feature reproducibility assessment, variance, and pairwise correlation analyses. First of all, only stable features should be included. For this reason, a preliminary analysis of multiple phantom acquisitions on both the same and different scanners should be conducted, in order to assess feature repeatability and reproducibility through the concordance correlation coefficient (CCC) and dynamic range (DR) calculation for each feature [14,45,46]. Subsequently, multiple segmentations by different physicians, algorithms, or software should be obtained to estimate the intraclass correlation coefficient (ICC) [46]. In summary, only those parameters that meet predetermined criteria (e.g., CCC and DR > 0.9, ICC > 0.75) can be considered stable and thus included in the analysis. After excluding unstable parameters, a further recommended step aims to remove features with low variance (presenting similar values in all samples), setting a variance threshold (usually 0.1).

Finally, additional unsupervised methods consist of eliminating redundant features. Indeed, redundancy within the information provided by different radiomic features is somewhat expected, mostly due to the great dimensionality. For this purpose, it may be useful to conduct a pairwise correlation analysis (variables that have a linear relationship with each other) and then eliminate highly colinear variables, leaving only one representative feature. Conversely, supervised techniques take into account the output variable when removing irrelevant features. These include wrapper and filter methods. Wrapper methods assess multiple models by adding and/or removing features to achieve the best-performing combination through a ranking approach (e.g., recursive feature elimination). By contrast, filter methods score the relationship between each feature and the target variable external to the predictive models. Only features with higher scores are then employed in the model (i.e., features are filtered based on their score). In addition to the wrapper and filter methods, some ML algorithms perform intrinsic feature selection naturally excluding non-informative input variables during training (e.g., tree- and rule-based models such as LASSO regression). Finally, an alternative to feature selection is represented by dimensionality reduction, which reduces the number of input variables in the training data, projecting them into a lower-dimensional feature space, de facto generating new input features.

There is no universal best feature selection method, set of input variables, or ML model. The best combination is actually the one that performs better in solving the specific problem under consideration. For this reason, the feature selection step is difficult to standardize and is usually empirically handled by the researcher with more or less systematic experiments, making it difficult to objectively evaluate the adopted approach. This represents further support for the importance of validating the results of a radiomic analysis, and whatever approach is taken for feature selection, it is key to accurately describe the process and possibly share the data. In this way, more researchers would be able both to repeat the experiments and implement the pipeline in new ones, with the potential of achieving better results.

On a final note, it is appropriate to highlight that all the above-mentioned steps can be intrinsically included in the hidden layers of DL-based classification approaches.

## 5. Data Processing for Model Training

Data preprocessing can be used to define the set of those steps that may be important to ensure or enhance the final performance and reliability of a radiomic model. Indeed, there are numerous issues that should be addressed with the manipulation of the selected data before the actual model training, including feature scaling as well as dealing with missing values or class imbalance [47]. The rationale behind the need for feature scaling is that features with a much greater variance than others, due to their wider value range, might appear dominant and greatly informative regardless of their actual value and overshadow the relevance of other features, thus biasing the learning process of a classifier [48]. Feature scaling can be achieved by the normalization or standardization of values.

Regarding missing values, it is unfortunately a possible occurrence that might involve data both in terms of clinical (e.g., specific laboratory test not available) and radiomic features (e.g., due to varying ROI size) [49]. While one could argue that a rigorous approach to the issue could be to entirely drop the instances with one or more missing values from the dataset, this would be rather undesirable in the clinical setting, where some values might be missing, but there would still be the need to use the predictive model. In this light, a different solution is represented by imputing the missing values, which can be accomplished using simple statistics (i.e., substituting the missing values with the mean, mode, or median values) or more complex multivariate modeling.

Class imbalance refers to the marked prevalence in a dataset of a particular class (in the set of a two-class dataset, we would have a majority and a minority class, the latter often being the one of interest for the classification purpose). This skewness in class distribution introduces a bias that might make it more difficult for the algorithm to accurately model the data distribution and thus lead to unreliable and poor predictive performance. There are two main strategies for dealing with class imbalance, namely undersampling and oversampling. A basic approach to undersampling would be to randomly discard a sufficient number of instances from the majority class to obtain a balanced dataset. Despite being a conceptually reasonable solution that could be easily applied to very large datasets, it is not feasible in the setting of radiology where the number of available instances (e.g., imaging exams, patients) is frequently limited. As a consequence, oversampling is more commonly adopted to address the class imbalance. Oversampling requires generating new instances, which can be carried out by randomly duplicating some of the instances available in the minority class or using more sophisticated techniques such as the synthetic minority oversampling technique (SMOTE) [50]. Briefly, SMOTE is a computational method that can be used to generate virtual instances from real ones. In the setting of DL, data augmentation can be also considered a valid alternative to solve the class imbalance. It is a technique that allows for an increase in the number of instances by adding slightly modified (e.g., mirrored, rotated) copies of the available medical images.

While handling the dataset before model training, it is of paramount importance to avoid information leakage, which is a well-known issue significantly contributing to the so-called reproducibility crisis in ML, involving and going far beyond the applications to oncologic imaging [51]. Data leakage occurs when there is not a clear separation between training and test datasets, which should instead be ensured in all imaging and data preprocessing, modeling, and validation steps. For example, the use of SMOTE prior to separating the training and test data represents a clear case of information leakage between the two sets, as the synthetic cases will include the information obtained from both.

## 6. Validation, Calibration, and Reporting Accuracy

**Figure 3:**
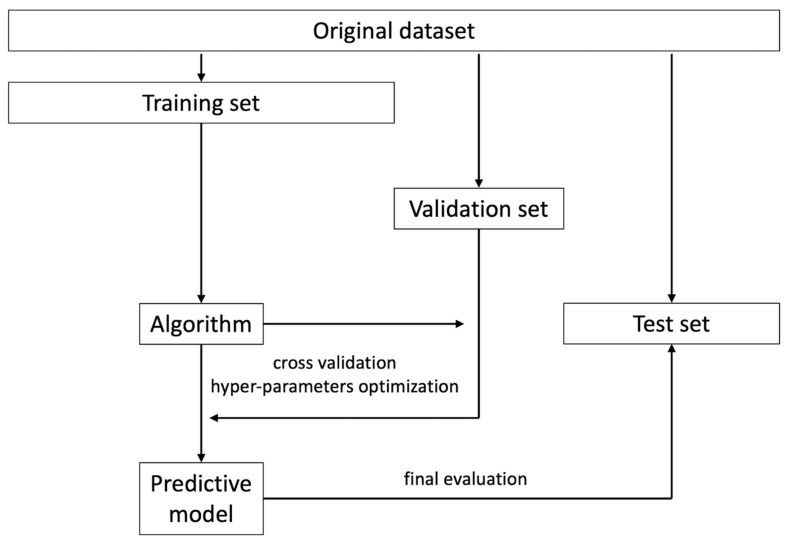
A flow diagram depicting the process of model training, validation, and testing using a single-institution dataset with holdout.

The concept of model “validation” within a radiomic and/or ML analysis pipeline may lead to some confusion for those readers who do not have firsthand experience in this type of work. Specifically, some papers seem to use the terms “validation” and “test” set interchangeably, when referring to data partitioning or collection, in addition to the more intuitively named training dataset. In actuality, the first two datasets both represent a distinct cohort from the training data, but they are employed for different aims and at different times within the pipeline (Figure 3) [52].

When building a model, it is generally fit using the training data alone. However, most types of ML algorithms have one or more settings (i.e., hyperparameters) that may be modified, affecting the resulting model’s performance. Similarly, the prior steps within the pipeline (e.g., image or data preprocessing, feature selection) may also vary, also influencing the final model’s accuracy of new data. The selection of optimal pipeline and model hyperparameter configuration requires a dataset that is clearly distinct from the training one, which is commonly referred to as a validation set. However, due to the iterative nature of the tuning process, the risk of introducing bias and overfitting the model to these data increases over time. Following this reasoning, it becomes apparent that, once the optimal pipeline is identified, a new batch of data is required to provide an unbiased estimation of the model’s performance in future cases. This is usually referred to as a test set, which may originate from the same data source (internal test set) or from a different one (external test set). The latter has a lower risk of bias and usually provides better insights into real-world model performance [53]. Obviously, modifying the model after the last step represents a clear pitfall and would require further testing on new data to avoid reporting biased results.

Several strategies have been proposed to reduce the amount of bias within the validation process, which would improve the final model’s performance on the test set. These have mainly been centered on data resampling, which can allow for more robust results, as the model evaluation is not limited to a single fitting and testing but to multiple such tests. The most common approaches are represented by resampling with replacement (i.e., bootstrapping) or without (e.g., k-fold cross-validation). In the first case, “novel” datasets are produced, with a variable number of “copies” of each original training instance (from none to multiple). Using cross-validation, the training data are split into k number of groups (i.e., folds). Then, one-fold is employed for the validation of a model fitted on the remaining ones, repeating the process for all k folds. Several variations exist with different values for k (e.g., 3, 5, 10, n-1), approaches for splitting the data folds (e.g., stratified, or randomized), and the number of iterations for the overall cross-validation process (i.e., one or more). Both bootstrapping and k-fold cross-validation have been employed in the setting of radiomics [40,41], and allow, for example, for the estimation of model test error.

The awareness of the value of predictive model calibration is starting to grow but is still less than ideal [54]. The focus on output simplification, a tendency to simplify outputs to binary labels, to accommodate the use of ML algorithms has led to a loss of nuance and tends to shift the decision-making process from the user (i.e., physician) onto the model [55,56]. Calibration statistics instead present a “goodness of fit” for the final model, which indicates the degree of agreement for predictions in relation to outcomes [57]. This can be achieved with a single score, such as the Brier score [58], through graphical representation (calibration plots), or with specific tests (e.g., the Hosmer–Lemeshow test) [59]. Currently, the inclusion of calibration statistics is becoming more common in radiomic studies [60,61,62,63], and their use is recognized as contributing to the methodological quality of an investigation [64].

Almost always, discriminative statistics are reported when describing the results of ML or statistical models built using radiomic features. These are related to the ability to correctly identify patients with a specific outcome, which, as stated above, is typically binary in the medical imaging applications of ML. Most physicians are fairly familiar with these accuracy metrics, which include the area under the receiver operating characteristic curve (AUC), sensibility, specificity, and negative and positive predictive values [57]. To better contextualize these values, it is ideal to also present the actual confusion matrix obtained from applying the model’s predictions to the unbiased test data. As with calibration statistics, thorough reporting of discrimination accuracy metrics, better if paired with resampling methods and corresponding confidence intervals, is a hallmark of good study methodological quality [64]. We must consider that, typically, one of these discriminative statistics is selected to guide the pipeline and model hyperparameter tuning process, which is usually aimed at minimizing its error rate. In turn, it becomes clear that the selection of the “best” metric must be based on the clinical context and prior domain knowledge of the clinical setting where the model is expected to be deployed. For instance, the overall accuracy (i.e., the ratio between the correctly classified and overall number of cases) may be severely misleading in data with a highly unbalanced class distribution [65]. Therefore, physicians must become more involved in model production, as this is the only way to overcome the current clinical translation issues of radiomic research efforts and develop the skills required to properly manage ML tools in daily practice [66,67].

## 7. Quality Evaluation Tools

**Figure 4:**
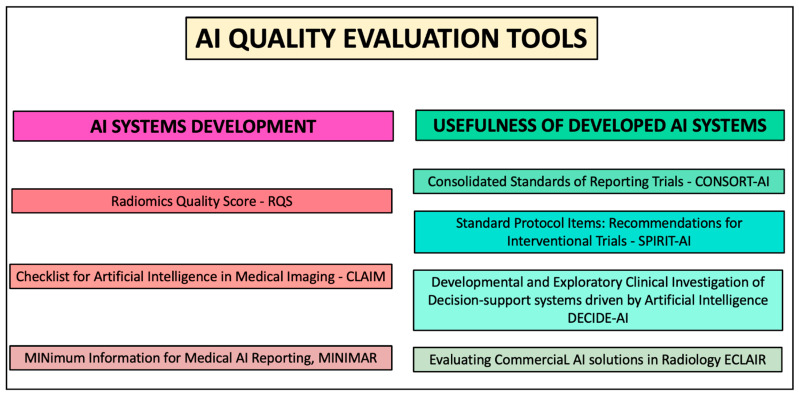
An overview of the main AI quality evaluation tools.

The growing interest in artificial intelligence (AI) and radiomic applications over the last few years, along with physicians’ relatively limited experience and knowledge of ML/DL methods, brought about the need for AI and radiomic guidelines to introduce researchers and clinical practitioners to these new techniques. Such recommendations could also be useful for those reviewers tasked to revise AI papers, providing them with the necessary instruments to identify drawbacks in the study methodology. Thus far, different documents have been produced for this purpose (Figure 4), which can be divided into instruments to conduct and evaluate AI studies prior to or after publication (e.g., Radiomic Quality Score, RQS, Checklist for Artificial Intelligence in Medical Imaging, CLAIM, and Minimum Information for Medical AI Reporting (MINIMAR)) [64,68,69], and tools to assess the usefulness of the already available AI software in a real-world setting, such as in the context of clinical trials (e.g., Standard Protocol Items: Recommendations for Interventional Trials—Artificial Intelligence (SPIRIT–AI) and Consolidated Standards of Reporting Trials—Artificial Intelligence (CONSORT–AI)) [70,71], in combination with human assessment (e.g., Developmental and Exploratory Clinical Investigation of Decision-support systems driven by Artificial Intelligence (DECIDE–AI)) [72] or when considering the adoption of commercial solutions (e.g., Evaluating Commercial AI solutions in Radiology (ECLAIR)) [73].

The RQS was released in 2017 and aimed to assess the radiomic workflow quality in different steps, from image protocol to feature calculation/selection, model building, and validation, as well as cost-effectiveness analysis [64]. Each step is assigned a score, for a cumulative quantitative assessment in terms of percentage that can be used as an effective appraisal tool for radiomic studies. In 2020, two additional checklists were proposed to illustrate the steps of a “best AI practice”, both from USA groups: CLAIM [68] and MINIMAR [69]. These are made up of different items, including those features that have to be considered for conducting a high-quality AI investigation, from study population to model building. Both checklists highlight the need for independent datasets to train and validate the proposed model, respectively. The MINIMAR guidelines also present some examples of how the required information should be reported in the paper. Further guidelines are currently being produced, such as The Transparent Reporting of a Multivariable Prediction Model of Individual Prognosis or Diagnosis (TRIPOD)–AI and the Prediction model Risk of Bias Assessment Tool (PROBAST)–AI, within the EQUATOR framework [74].

AI tools are meant to be used in clinical practice, so the inclusion of a developed AI algorithm in clinical trials appears crucial to fully assess their applicability. In this light, the SPIRIT and CONSORT guidelines have been integrated with specific recommendations related to the employed AI software in clinical trials (SPIRIT–AI and CONSORT–AI), such as name/version, image acquisition protocol, and information on how the software was used (e.g., alone or in combination with clinical data) [70,71]. A similar initiative was carried out by the DECIDE–AI expert group, to provide recommendations and guidelines for the early stage clinical evaluation of decision support systems using a Delphi approach. The resulting checklist includes indications on how to build an investigation for the assessment of the usefulness of an AI tool in clinical practice, such as the description of the patient population, features of the employed AI system, or how the system interacted with human evaluation [72]. At present, several AI tools have been proposed for clinical use, with the majority for neuro applications [75]. However, only a minority of such systems proved to have a high level of evidence validating the impact on clinical practice, patient outcomes, and costs [76]. To aid the physicians interested in potentially adopting these commercially available products in their institution, the ECLAIR guidelines provide a set of 20 questions that should be considered prior to purchase [73]. These are divided into several domains, including regulatory, legal, and financial aspects, which become particularly relevant when translating radiomics and AI from a research setting to potentially affecting patient outcomes in daily clinical practice.

Although a high heterogeneity still exists in terms of the applied AI methods, the current efforts to standardize AI techniques and provide shared guidance on how to conduct, report, and evaluate AI investigations will lead to the production of more robust evidence on AI efficacy and clinical implementation.

## 8. Further Considerations

There are some additional aspects worth mentioning that should be taken into account when designing a radiomic study. For instance, in the era of big data, radiomics should be considered a part of a vastly greater world of “-omics” and, as a consequence, predictive models should aim to integrate all sources of clinically relevant data in a comprehensive manner (holomics) [77]. Whenever feasible, non-radiomic features (e.g., data from clinical records, or biological or genetic sources) should be incorporated into more holistic models. This might, for example, facilitate the identification of biological correlates and help create a connection between imaging and genomics, as it happens in the field of radiogenomics [78].

When presenting a radiomic model, one should consider what might be the actual clinical implication of its medical use. A formal assessment of the potential clinical relevance of radiomic models can be made by using decision curve analysis [79]. However, one might also want to provide a comparison of the model’s performance to that of the current gold standard. The benefits of radiological workflows could also be explored, for example, in terms of reduced reading time and increased accuracy and consistency among radiologists.

## 9. Conclusions

While the future of radiomics still looks bright, unmet methodological challenges are casting worrying shadows. The outcome for oncologic patients would benefit from more precise, tailored approaches, and radiomics might assist physicians in taking one more step in this direction. However, to which extent radiomics will redefine oncologic patients’ management remains to be seen and will require robust, reproducible evidence built on state-of-the-art pipelines.

## Figures and Tables

**Table 1:** Image preprocessing techniques are presented, with their rationale and advantages.

Image Preprocessing Technique	Rationale	Advantage
**Normalization**	MRI data contain arbitrary intensity units and grey-level intensity that can be homogenized with intensity outlier filtering (e.g., calculating the mean and standard deviation of grey levels and excluding those outside a definite range such as mean ± 3 times the standard variation).	Reducing the heterogeneity due to varying pixel grey-level value distribution across exams
**Resampling**	Images with different spatial resolutions can be uniformed and either upscaled or downscaled to isotropic voxel spacing.	Increases reproducibility by making texture features rotationally invariant
**Discretization**	Grouping pixels into bins based on intensity ranges, which is conceptually similar to creating a histogram.	A greater number of bins (or a smaller bin width) tend to preserve image details at the cost of noise. Conversely, noise reduction can be achieved by reducing the number of bins (or increasing bin width) but will cause the image to lose detail.
**Bias field correction**	MRI can suffer from spatial signal variation caused by the magnetic field being intrinsically inhomogeneous.	Correct undesired inhomogeneities
**Image filtering**	Application of edge enhancing (e.g., Laplacian of Gaussian) or decomposition (e.g., wavelet transform) filters to obtain additional image volumes from which to extract features.	May emphasize useful image characteristics while reducing noise

## Data Availability

Not applicable.
